# Anomalous weak values via a single photon detection

**DOI:** 10.1038/s41377-021-00539-0

**Published:** 2021-05-25

**Authors:** Enrico Rebufello, Fabrizio Piacentini, Alessio Avella, Muriel A. de Souza, Marco Gramegna, Jan Dziewior, Eliahu Cohen, Lev Vaidman, Ivo Pietro Degiovanni, Marco Genovese

**Affiliations:** 1 INRIM, Strada delle Cacce 91, I-10135 Torino, Italy; 2 National Institute of Metrology, Quality and Technology—INMETRO, Av. Nossa Senhora das Graças, 50, Duque de Caxias, RJ 25250-020 Brazil; 3 Max-Planck-Institut für Quantenoptik, Hans-Kopfermann-Straße 1, 85748 Garching, Germany; 4 Department für Physik, Ludwig-Maximilians-Universität, 80797 München, Germany; 5 Faculty of Engineering and the Institute of Nanotechnology and Advanced Materials, Bar Ilan University, Ramat Gan, 5290002 Israel; 6 Raymond and Beverly Sackler School of Physics and Astronomy, Tel-Aviv University, Tel-Aviv, 6997801 Israel

**Keywords:** Quantum optics, Single photons and quantum effects

## Abstract

Is it possible that a measurement of a spin component of a spin-1/2 particle yields the value 100? In 1988 Aharonov, Albert and Vaidman argued that upon pre- and postselection of particular spin states, weakening the coupling of a standard measurement procedure ensures this paradoxical result^1^. This theoretical prediction, called *weak value*, was realised in numerous experiments^2–9^, but its meaning remains very controversial^10–19^, since its “anomalous” nature, i.e., the possibility to exceed the eigenvalue spectrum, as well as its “quantumness” are debated^20–22^. We address these questions by presenting the first experiment measuring anomalous weak values with just a single click, without the need for statistical averaging. The measurement uncertainty is significantly smaller than the gap between the measured weak value and the nearest eigenvalue. Beyond clarifying the meaning of weak values, demonstrating their non-statistical, single-particle nature, this result represents a breakthrough in understanding the foundations of quantum measurement, showing unprecedented measurement capability for further applications of weak values to quantum photonics.

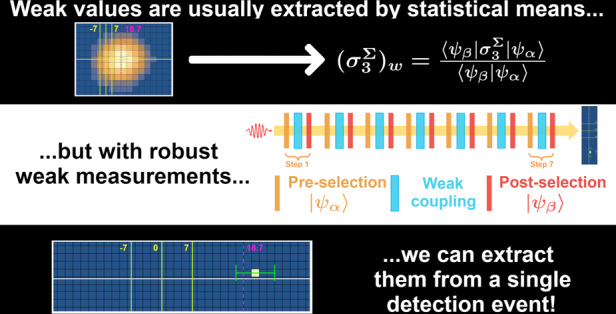

Weak values (WVs), as introduced in ref. ^1^, represent one of the most interesting and intriguing quantum measurement paradigms. In that influential work, the outcome of the measurement of the spin component was 100, while in the chosen units its maximal eigenvalue was just 1. However, weakening the coupling in the measurement procedure made the uncertainty in an individual measurement much larger than 1 (and even than 100), thus this “anomalous” value was observed only after averaging over a very large number of readings of the pointer variable. While averaging is a standard practice in many measurement protocols, postselection is not, hence the legitimacy of the statistical analysis was questioned^20–22^. Understanding this matter is fundamental not only for clarifying the meaning of WVs, but also in view of significant applications in quantum metrology^3–5,23,24^. In this work, we present a *robust weak measurement*, an experiment in which a single reading of the measuring device, coupled to the system only once, provides a WV and, in particular, an anomalous one. Postselection still plays a crucial role, but the anomalous outcome no longer arises from a statistical analysis. We measured an observable with eigenvalues in the range [−7, 7]. The WV of the observable of the pre- and postselected system on which a single-click measurement was performed was 18.7, and our single click yielded 21.4 ± 4.5, see Fig. 1.

**Fig. 1:**
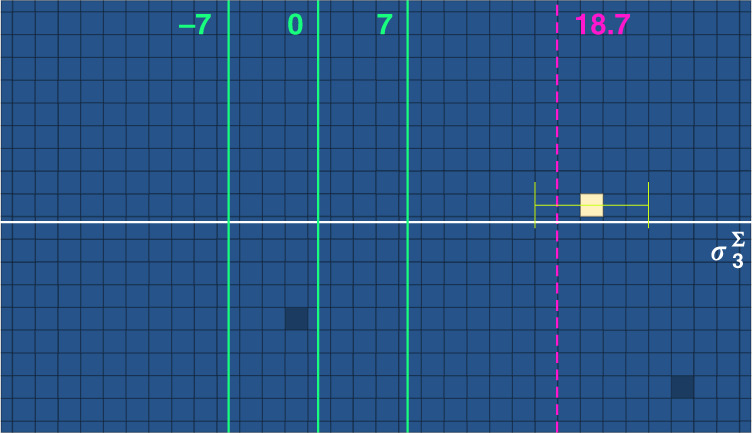
Single detection event yielding an anomalous weak value of ${\sigma }_{3}^{{{\Sigma }}}$. The vertical solid lines show the borders and centre of the eigenvalue spectrum of our observable, while the dashed line indicates its weak value calculated according to the experimental parameters, i.e., ${({\sigma }_{3}^{{{\Sigma }}})}_{w}=18.7$. The experimental point, shown in white, gives the value ${({\sigma }_{3}^{{{\Sigma }}})}_{w}^{{\rm{1}}\ {\rm{click}}}=21.4$. The uncertainty, represented by the horizontal green bars, is specified by calculating the width of the spatial wave function of the quantum particle before the detection, and confirmed by repeating the experiment many times

This is a surprising result, since the expectation value of the observable in the preselected state was only 2.2. It would not be surprising that postselection on the eigenstate corresponding to a maximal eigenvalue (i.e., 7) slightly increased the measured value, but only up to 7, not beyond (in fact, in our experiment the expectation value corresponding to the postselected state was also just 2.2).

The main theoretical basis for our experiment is the work in ref. ^25^, which preceded the introduction of WVs^1^, extended here by constructing a new method allowing a feasible experimental implementation. The easiest way to explain this work is via weak measurements performed on a system consisting of *n* particles with a single measuring device, see Sec. VII of ref. ^26^. Within the standard weak measurement procedure, in which each particle has its own measuring device, the uncertainty of measuring the sum of variables ${A}^{{{\Sigma }}}\equiv \mathop{\sum }\nolimits_{k = 1}^{n}{A}_{k}$, where *A*_*k*_ corresponds to particle *k*, scales like $\sqrt{n}$ because of the contributions of the *n* measuring devices. In our procedure for measuring *A*^Σ^, we couple a single measuring device to all particles and thus, importantly, the final uncertainty is the one of the single measuring device involved, avoiding the $\sqrt{n}$ increase. This allows extracting an anomalous WV ${({A}^{{{\Sigma }}})}_{w}$ by coupling the measuring device only once to each part of the system, and observing only a single click of the measuring device.

In our experimental demonstration, the measured variable is the sum of polarisation variables of *n* photons:

1 $${\sigma }_{3}^{{{\Sigma }}}\equiv \mathop{\sum }\limits_{k=1}^{n}{\sigma }_{3}^{(k)}$$

where ${\sigma }_{3}\equiv \left|H\right\rangle \langle H| -| V\rangle \left\langle V\right|$, and *H*(*V*) is the horizontal (vertical) polarisation. All photons are preselected in the state

2 $$\left|{\psi }_{\alpha }\right\rangle =\cos \alpha \left|H\right\rangle +\sin \alpha \left|V\right\rangle$$

and postselected in the state

3 $$|{\psi }_{\beta }\rangle =\cos \beta |H\rangle +\sin \beta |V\rangle$$

When states (2) and (3) are almost orthogonal, we obtain an anomalously large weak value

4 $${\left({\sigma }_{3}^{{{\Sigma }}}\right)}_{w}=\frac{\langle {\psi }_{\beta }| {\sigma }_{3}^{{{\Sigma }}}| {\psi }_{\alpha }\rangle }{\langle {\psi }_{\beta }| {\psi }_{\alpha }\rangle }$$

All photons are coupled to the same measuring device, so the interaction Hamiltonian is

5 $${\mathcal{H}}=g(t)\ \mathop{\sum }\limits_{k = 1}^{n}{\sigma }_{3}^{(k)}\otimes {p}_{x}$$

where a well localized *g*(*t*) defines the time of the measurement, ∫*g*(*t*) d*t* = 1, and *p*_*x*_ is the conjugate momentum of the pointer variable *x* with the initial quantum state modelled by the Gaussian

6 $$\chi (x)=\frac{1}{\sqrt{{{\Delta }}\sqrt{2\pi }}}{e}^{-{x}^{2}/4{{{\Delta }}}^{2}}$$

The strength of the measurement interaction in Eq. (5) is characterized by $\frac{1}{{{\Delta }}}$, which has to be chosen carefully: for a single-click measurement the uncertainty cannot be too large, but also the coupling cannot be too strong, as it changes the WV^19,27^. Moreover, although the system has a well-defined WV of the measured observable at every moment between pre- and postselection, the measuring device, in general, does not indicate this WV, because of entanglement between system and measuring device, see Fig. 5 in ref. ^22^. Somewhat surprisingly, in the case of coupling to a Gaussian pointer, the expectation value of the pointer after the measurement exactly equals this WV. Since, in the present scenario, the WV is constant in time during the measurement interaction, we can extract it at the moment just before the postselection, by calculating the quantum state after the interaction and knowing the postselection state.

To make the explanation more transparent, let us first consider a case for *n* = 1. The state of the photon and the measuring device before the postselection is

7 $$\cos \alpha \left|H\right\rangle |{\chi}_{+}\rangle +\sin \alpha \left|V\right\rangle |{\chi}_{-}\rangle$$

where $|{\chi }_{+}\rangle$ and $|{\chi }_{-}\rangle$ denote Gaussians shifted by 1 and −1, respectively. Thus, the photon has a mixed polarisation state described by the density matrix *ρ*_*α*_, that can be expressed in the $\left\{\left|H\right\rangle ,\left|V\right\rangle \right\}$ basis as

8 $${\rho }_{\alpha }=\left(\begin{array}{ll}{\cos }^{2}\alpha &{e}^{-\frac{1}{2{{{\Delta }}}^{2}}}\sin \alpha \cos \alpha \\ {e}^{-\frac{1}{2{{{\Delta }}}^{2}}}\sin \alpha \cos \alpha &{\sin }^{2}\alpha \end{array}\right)$$

Postselecting on $|{\psi }_{\beta }\rangle$, the WV is given by (see Eq. (32) in ref. ^22^)

9 $${({\sigma }_{3})}_{w}=\frac{{\rm{tr}}(|{\psi }_{\beta }\rangle \langle {\psi }_{\beta }|{\sigma }_{3}{\rho }_{\alpha })}{{\rm{tr}}(|{\psi }_{\beta }\rangle \langle {\psi }_{\beta }|{\rho }_{\alpha })}=\frac{{\mu }^{2}-{\nu }^{2}}{{\mu }^{2}+{\nu }^{2}+2\mu \nu {e}^{-\frac{1}{2{{{\Delta }}}^{2}}}}$$

where $$\mu =\cos \alpha \cos \beta$$ and $$\nu =\sin \alpha \sin \beta$$.

It is straightforward to generalise the calculation for *n* > 1. We define the joint pre- and postselected states of the *n* photons as, respectively, $\left|{{{\Psi }}}_{\alpha }\right\rangle \,\,{ = \bigotimes }_{k = 1}^{n}|{\psi }_{\alpha }^{(k)}\rangle$ and $\left|{{{\Psi }}}_{\beta }\right\rangle\,\, { = \bigotimes }_{k = 1}^{n}|{\psi }_{\beta }^{(k)}\rangle$. The WV is

10 $$\begin{array}{lll}{\left({\sigma}_{3}^{{{\Sigma}}}\right)}_{w}&=&\frac{\left\langle{{{\Psi}}}_{\beta}\right| {\sigma}_{3}^{{{\Sigma}}}\,{\rm{tr}}\left({U}^{{{\Sigma}}}\left| {{{\Psi}}}_{\alpha}\right\rangle\langle {{{\Psi}}}_{\alpha}| \otimes | \chi \rangle \langle \chi | {\left({U}^{{{\Sigma}}}\right)}^{\dagger}\right)\left| {{{\Psi}}}_{\beta}\right\rangle}{\langle {{{\Psi}}}_{\beta}| {\rm{tr}}\left({U}^{{{\Sigma}}}| {{{\Psi}}}_{\alpha}\rangle \langle {{{\Psi}}}_{\alpha}| \otimes | \chi \rangle \langle \chi | {\left({U}^{{{\Sigma}}}\right)}^{\dagger}\right)| {{{\Psi}}}_{\beta}\rangle}\\ &=&\frac{\mathop{\sum}\nolimits_{k,l = 0}^{n}\left({{n}\atop{k}}\right)\left({{n}\atop{l}}\right){\mu}^{k+l}{\nu}^{2n-k-l}\left(2k-n\right){\gamma}_{kl}}{\mathop{\sum}\nolimits_{k,l = 0}^{n}\left({{{n}}\atop{{k}}}\right)\left({{n}\atop{l}}\right){\mu}^{k+l}{\nu}^{2n-k-l}{\gamma}_{kl}}\end{array}$$

where ${U}^{{{\Sigma }}}={e}^{-i\mathop{\sum }\nolimits_{k = 1}^{n}{\sigma }_{3}^{(k)}\otimes {p}_{x}}$, ${\gamma }_{kl}={e}^{-\frac{{(k-l)}^{2}}{2{{{\Delta }}}^{2}}}$, and the trace is taken over the pointer system only. Our Gaussian pointer, after the measurement, shows $\langle x\rangle ={\left({\sigma }_{3}^{{{\Sigma }}}\right)}_{w}$ with uncertainty ${{\Delta }}x=\sqrt{\langle {x}^{2}\rangle -{\langle x\rangle }^{2}}$, where

11 $$\langle {x}^{2}\rangle =\frac{\mathop{\sum }\nolimits_{k,l = 0}^{n}\left({n}\atop{k}\right)\left({n}\atop{l}\right){\mu }^{k+l}{\nu }^{2n-k-l}\left({(n-k-l)}^{2}+{{{\Delta }}}^{2}\right){\gamma }_{kl}}{\mathop{\sum }\nolimits_{k,l = 0}^{n}\left({n}\atop{k}\right)\left({n}\atop{l}\right){\mu}^{k+l}{\nu }^{2n-k-l}{\gamma }_{kl}}$$

The experiment directly testing this prediction, see Fig. 2a, is very difficult.

**Fig. 2:**
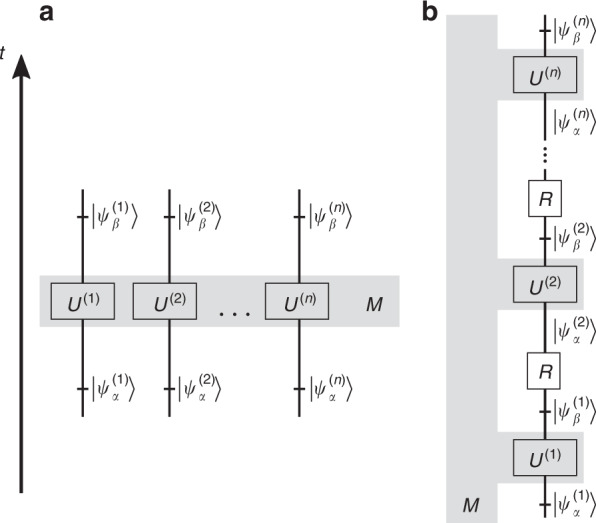
Robust weak measurement: theoretical framework. **a** A measuring device *M* is coupled simultaneously to *n* particles of a pre- and postselected system. **b** The measuring device is coupled to the same particle at *n* times with particular pre- and postselection at each time. After each postselection onto $|{\psi }_{\beta }^{(k)}\rangle$, a unitary rotation *R* restores the preselection state $\left|{\psi }_{\alpha }\right\rangle$

The following observation leads to a more feasible test of our predictions. In the Hamiltonian in Eq. (5) the variables ${\sigma }_{3}^{(k)}$ are constants of motion, therefore it does not matter when the interaction with every photon takes place. In particular, we can perform the couplings one after another. In this case, however, we can use just one photon instead of *n*, pre- and postselecting it *n* times according to the states in Eqs. (2) and (3), see Fig. 2b. The observable, i.e., the sum of polarisation variables of *n* photons, is replaced by the sum of polarisation variables of the same photon at *n* different times, which is much easier to measure, since the spatial degree of freedom of this single photon can play the role of the measuring device.

We consider the case *n* = 7, which is large enough to see the predicted effect. In Fig. 3 we show the WV ${({\sigma }_{3}^{{{\Sigma }}})}_{w}$ and the pointer uncertainty Δ*x* (Eqs. (10) and (11), respectively) for *α* = 0.62 and Δ = 5.84, as a function of *β*. By choosing *β* = 2.53, one obtains a theoretical final state of the pointer showing 18.7 ± 4.5, granting an optimal reading of the anomalous WV in terms of relative uncertainty. Note that the final width of the pointer is smaller than the initial width, due to a subtle narrowing effect^28^.

**Fig. 3:**
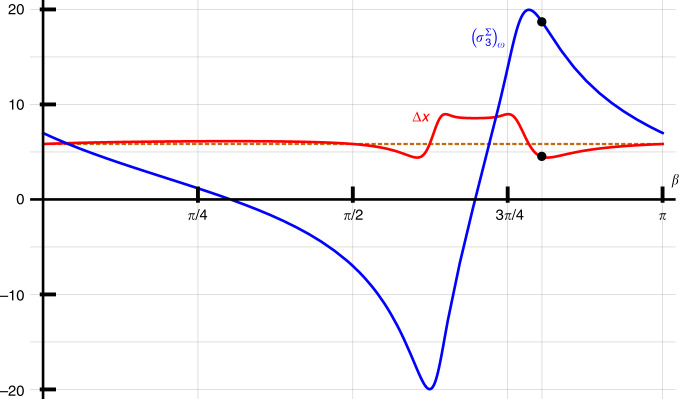
Predicted weak value and pointer uncertainty. Predicted values for the WV ${({\sigma }_{3}^{{{\Sigma }}})}_{w}$ (solid blue line) and the final pointer uncertainty Δ*x* (solid red line) for *n* = 7, *α* = 0.62 and Δ = 5.84, as a function of *β*. The initial photon distribution width Δ is included as a dashed brown line. The two black dots on the curves denote the ${({\sigma }_{3}^{{{\Sigma }}})}_{w}=18.7$ and Δ*x* = 4.5 obtained for *β* = 2.53, the parameters chosen for our experimental demonstration

The experimental setup is shown in Fig. 4. It is composed of a set of *n* = 7 blocks in which a birefringent crystal pair realises the weak interaction, preceded by a half-wave plate and followed by a polarising plate. While the polarising plate performs the postselection, the half-wave plate rotates the polarisation of the photon outgoing the previous block to set the preselection state. The EM-CCD placed at the end of the *n* = 7 blocks detects the arrival position of the photon. A detailed description of the setup is provided in the Methods section.

**Fig. 4:**
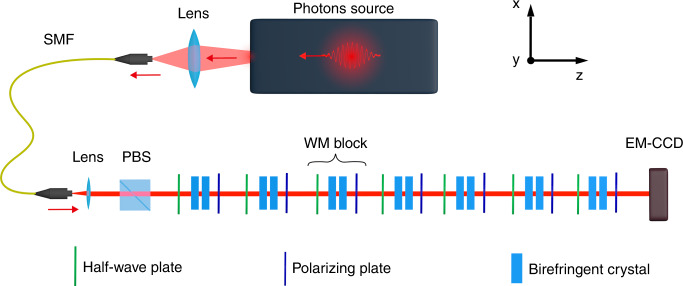
Experimental setup. Our photon source exploits type-I spontaneous parametric down conversion. Generated signal photons at 702 nm are spectrally-filtered, injected in a single-mode fibre and then collimated in a Gaussian mode to be used in the experiment, while idler photons at 920 nm are detected by a Single-Photon Avalanche Detector in order to monitor the stability of the source. The robust weak measurement is obtained by means of the *n* = 7 identical blocks put after the initial PBS. A spatially-resolving detector (EM-CCD camera operating in the photon counting regime) is used to determine the final position of the detected photons

The single-click experiment presented in Fig. 1 yielded 21.4, in agreement with the predicted value within the theoretical uncertainty.

We tested our theoretical analysis a posteriori by performing a multi-click experimental run with the same parameters *α*, *β* and Δ, to record the distribution shown in Fig. 5. The mean value of this multi-click distribution, 18.59, is very close to the theoretical WV ${({\sigma }_{3}^{{{\Sigma }}})}_{w}=18.7$, given by Eq. (10). The statistical uncertainty is only 0.09, thus the remaining discrepancy is due to imprecision in the parameters *α*, *β*, Δ fixing the theoretical value. Some deviations are caused by imperfections in the optical elements, e.g., the birefringent crystals (details in the Supplementary Material). The width of the multi-click distribution in Fig. 5 turns out to be 4.5, in full agreement with the theoretical predictions.

**Fig. 5:**
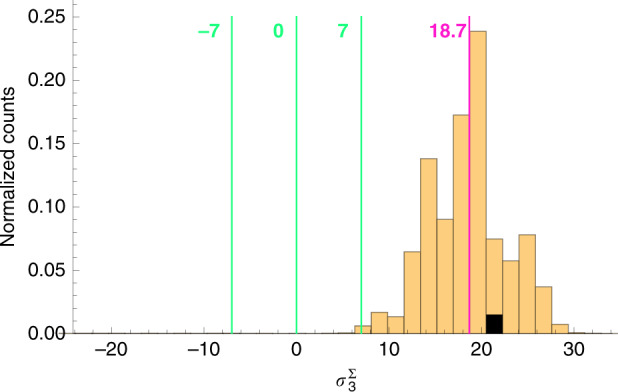
Measurement of anomalous weak value. Normalized histogram of the photon counts along the *x* axis of the EM-CCD (see Methods) for repetitions of the single-click experiment (with unchanged parameters). The black square indicates the first click of the run, corresponding to the single-click experiment. The green lines indicate the borders and centre of the eigenvalue spectrum of our observable. The purple line shows the expected (theoretical) weak value ${({\sigma }_{3}^{{{\Sigma }}})}_{w}$

As in all weak measurements in which the pointer variable of the polarisation measurement of a photon is its transverse degree of freedom, the probability distribution in Fig. 5 is identical to the intensity pattern of the electromagnetic wave on the screen which can be calculated using Maxwell equations (see refs. ^4,29^). This is a sufficiently narrow distribution to allow a robust measurement of the WV via the detection of a single quantum particle. In the framework of classical physics, this bizarre result is hidden in the peculiar interference phenomena of superoscillations^30,31^. Thus, the quantum formalism of robust weak values, apart from describing a genuinely quantum effect for the case of measurement with an external pointer, naturally incorporates this classical interference effect.

To increase confidence in our results, we repeated the experiment for a few sets of different parameters leading to less anomalous WVs (and even a non-anomalous one). All results are presented in Table 1 (see the Supplementary Material for additional information). The results are influenced by uncertainties originating from the calibration procedure. However, when the purpose is not to find a precise numerical value of the polarisation WV, but to test the single-click measurement method versus an ensemble measurement, the calibration uncertainty is irrelevant. The experimental data shown in Table 1 fit the theoretical predictions well, proving the possibility of measuring an anomalous WV with a single detection.

**Table 1:** Results for the various parameters of the measurement setup

#	1	2	3	4	5	6	7	8	9	10
	*α*	*β*	Δ	${({\sigma }_{3}^{{{\Sigma }}})}_{w}$	${{\Delta }}[{({\sigma }_{3}^{{{\Sigma }}})}_{w}]$	${({\sigma }_{3}^{{{\Sigma }}})}_{w}^{\,\text{exp}\,}$	${{{\Delta }}}_{\text{stat}}[{({\sigma }_{3}^{{{\Sigma }}})}_{w}^{\,\text{exp}\,}]$	${({\sigma }_{3}^{{{\Sigma }}})}_{w}^{\,\text{1 click}\,}$	${{\Delta }}{({\sigma }_{3}^{{{\Sigma }}})}_{w}^{\,\text{1 click}\,}$	Δ*x*
(a)	0.62	2.53	5.84	18.7	0.9	18.59	0.09	21.4	4.5	4.5
(b)	0.62	2.53	3.18	9.8	0.8	10.51	0.02	10.9	3.8	5.0
(c)	0.52	2.62	2.96	11.4	0.5	11.07	0.08	14.1	3.4	2.8
(d)	0.52	0.88	3.09	1.3	0.4	0.97	0.05	−2.6	4.6	3.6

Columns 1-4 describe the preparation parameters and the corresponding weak value ${({\sigma }_{3}^{{{\Sigma }}})}_{w}$. Column 5 shows the systematic uncertainty in the experimental implementation of ${({\sigma }_{3}^{{{\Sigma }}})}_{w}$ due to the uncertainties in the preparation parameters *α*, *β* and Δ (which incorporate inhomogeneities in birefringent crystals and other experimental imperfections). Column 6 shows the experimental mean values obtained by repeating the single-photon experiments (EM-CCD dark counts subtracted). The statistical uncertainty in these experiments is shown in column 7. Column 8 presents the experimental weak values ${({\sigma }_{3}^{{{\Sigma }}})}_{w}^{\,\text{1 click}\,}$ extracted from a single detection event. The uncertainty in column 9 represents the quantum uncertainty of the pointer variable experimentally obtained from repeated measurements with the same parameters as the single-click experiment (see histogram in Fig. 5). Column 10 contains the predicted final uncertainty of the pointer, Δ*x*, calculated from the parameters *α*, *β*, and Δ

Theoretical calculations, see Fig. 3, show that the final uncertainty of the pointer is close to the initial beam width Δ. Thus, faced with a new task of estimating ${\left({\sigma }_{3}^{{{\Sigma }}}\right)}_{w}$ in which somebody else fixes the pre- and postselected states of the system, a single click on our detector is capable of providing the WV with an uncertainty of the same order of magnitude as the width of the initial beam even for anomalous WVs.

In summary, our results offer a deeper understanding of the meaning of WVs, providing a significant contribution to the development of quantum measurement in the weak coupling regime. Estimating anomalous WVs with a single-click breaks the common belief that the WV is a statistical concept of conditional expectation value^13^. Our findings, going well beyond refs. ^22,32^, stress the non-statistical, single-particle nature of WVs, demonstrating how a single-click measurement can provide a WV estimate even for anomalous WVs. Furthermore, this experiment suggests a viable possibility for amplification methods effectively reducing the uncertainty contribution associated with the measurement of the pointer. This paves the way for future practical applications of the robust weak measurement paradigm.

## Methods

In our experimental setup, shown in Fig. 4 (further details in the Supplementary Material), photons in a multi-thermal distribution with a mean photon number per pulse ≪ 1 are produced by type-I spontaneous parametric down-conversion (SPDC). This guarantees a short coherence time (~150 fs), avoiding unwanted self-interference effects due to internal reflections. The SPDC occurs in a 10 × 10 × 5 mm LiIO_3_ crystal, pumped by the second harmonic generation (398 nm) of a 76 MHz mode-locked laser at 796 nm. The signal photons are spectrally filtered and coupled to a single-mode fibre. At the end of the fibre, the photons are collimated in a Gaussian mode and sent to the free-space path where the robust weak measurement experiment occurs. After passing through an initial polarising beam splitter (PBS), used to suppress any residual circular polarisation component, the signal photons go through *n* = 7 identical blocks, each implementing three steps: preselection, weak coupling and postselection. Each photon enters every block in a linear polarisation state, due to either the first PBS or the postselection of the previous block. Every block begins with a quartz half-wave plate, implementing a unitary rotation *R* aligning the photon polarisation axis to the direction corresponding to the initial (preselected) state $|{\psi }_{\alpha }\rangle$. Then, a birefringent unit composed of a pair of birefringent crystals is responsible for the weak interaction. The first calcite crystal, 2 mm long, has the extraordinary (*e*) optical axis lying in the *x* − *z* plane, forming an angle of *π*/4 with respect to the *z* direction. This generates a spatial walk-off (of ~0.2 mm) along the *x* direction for the horizontally-polarised photons, reducing the overlap between the horizontal and vertical polarisation components. The second crystal of each unit is a 1.1 mm long calcite crystal with the optical *e*-axis lying along the *y* direction. It generates no spatial walk-off, and its role is to compensate the temporal walk-off induced by the first crystal. The last component of each block is a polarising plate, postselecting the photons in the state $\vert{\psi }_{\beta }\rangle$. After the *n* = 7 blocks, the photons are detected by a spatially resolving detector, i.e., an Electron Multiplying CCD (EM-CCD) device able to work in the linear analogue regime as well as in the photon counting regime (details in ref. ^33^). To calibrate our system, we identify the extremal positions of the pointer on our EM-CCD, i.e. the position of the $\left|V\right\rangle$ polarisation state, corresponding to the eigenvalue ${\sigma }_{3}^{{{\Sigma }}}=-7$ and then the position of the state $\left|H\right\rangle$, corresponding to ${\sigma }_{3}^{{{\Sigma }}}=7$. This allows us to define the zero point as the centre between the readings for ${\sigma }_{3}^{{{\Sigma }}}=\pm 7$, and also scale the pointer variable accordingly.

## Supplementary information

Supplementary Information for:
