# Supplementary material for: Anomalous weak values via a single photon detection
Source: Light Sci Appl. 2021 May 25;10:106. doi: 10.1038/s41377-021-00539-0 (PMC8149841; doi:10.1038/s41377-021-00539-0)
Supplement: Supplementary file 1 — Supplementary Information for: [file 41377_2021_539_MOESM1_ESM.pdf]

Supplementary Information for: "Anomalous weak values via a single photon detection"

Enrico Rebufello[1], Fabrizio Piacentini[1], Alessio Avella[1], Muriel A. de Souza[2], Marco Gramegna[1], Jan Dziewior[3,4], Eliahu Cohen[5], Lev Vaidman[6], Ivo Pietro Degiovanni[1], Marco Genovese[1]

[1] *INRIM, Strada delle Cacce 91, I-10135 Torino, Italy*

[2] *National Institute of Metrology, Quality and Technology - INMETRO; Av. Nossa Senhora das Graças, 50, 25250-020, Duque de Caxias- RJ– Brazil*

[3] *Max-Planck-Institut für Quantenoptik, Hans-Kopfermann-Straße 1, 85748 Garching, Germany*

[4] *Department für Physik, Ludwig-Maximilians-Universität, 80797 München, Germany*

[5] *Faculty of Engineering and the Institute of Nanotechnology and Advanced Materials, Bar Ilan University, Ramat Gan 5290002, Israel*

[6] *Raymond and Beverly Sackler School of Physics and Astronomy, Tel-Aviv University, Tel-Aviv 6997801, Israel and*

* *To whom correspondence should be addressed; E-mail: f.piacentini@inrim.it*

I. DETAILS OF THE SETUP

Our experimental setup (scheme reported in the Methods section) hosts a 796 nm mode-locked Ti:Sapphire laser (repetition rate: 76 MHz), whose second harmonic emission (398 nm) pumps a $10 \times 10 \times 5$ mm $LiIO_3$ non-linear crystal where the Type-I Spontaneous Parametric Down-Conversion (SPDC) occurs. Idler photons ($\lambda_i = 920$ nm) are spectrally filtered by means of a low-loss custom interference filter (IF) with full-width at half maximum (FWHM) of 10 nm, coupled to a single-mode fiber (SMF) and then addressed to a silicon single-photon avalanche diode (Si-SPAD), in order to detect and evaluate eventual fluctuations in the SPDC process. Correlated signal photons ($\lambda_s = 702$ nm), after being spectrally filtered (again with a low-loss IF, FWHM = 20 nm) and SMF-coupled, are addressed to a launcher collimating them in a Gaussian mode in the free-space optical path where the robust weak measurement takes place.

In this path, after an initial state filtering stage in which a polariser prepares our signal photons in the horizontally-polarised state, one can find a sequence of $n = 7$ identical measurement stages, each implementing the preselection, weak interaction and postselection operation needed for the robust weak measurement. The Hamiltonian evolution of the quantum state is induced by exploiting birefringence. In our optical path we can insert up to $n = 7$ birefringent units, each of them composed of two different calcite crystals. The number of units was chosen considering the trade-off between the resolution of the system (i.e. the spatial separation between the minimum and maximum of the eigenvalue spectrum of our observable) and the success probability in case of strongly anomalous weak values, together with the losses originating from imperfections of the optical elements and the non-unit quantum efficiency and dark counts level of the detector. Further increasing n would result in an overall photon survival probability too small to grant a satisfactory signal-to-noise ratio at the detector output.

Each stage starts with a quartz half-wave plate (HWP), realising the unitary evolution R preparing the single photons in the preselected state $|\psi_\alpha\rangle = \cos\alpha|H\rangle + \sin\alpha|V\rangle$. Afterwards,

the weak interaction is mediated by a pair of birefringent crystals. The first one is a 2 mm long calcite crystal whose extraordinary (e) optical axis lies in the x-z plane, with an angle of $\pi/4$ with respect to the z direction. Due to the spatial walk-off effect experienced by the horizontally-polarised photons along the x direction, horizontal and vertical-polarisation paths get slightly separated. The second one is a 1.1 mm long calcite crystal with the optical e-axis orthogonal to the x direction (thus not generating spatial walk-off) used to nullify, through phase compensation, the temporal walk-off introduced by the first crystal. Each measurement stage ends with the postselection part, i.e. a linear polariser selecting the photons in the final state $|\psi_\beta\rangle = \cos\beta|H\rangle + \sin\beta|V\rangle$. Thanks to the short coherence time ($\sim$ 150 fs) of the down-converted photons, we can avoid interference effects due to reflections between adjacent optical surfaces. At the end of the optical path, the photons are detected by an Electron Multiplying CCD (EM-CCD) device able to work both in the linear analog regime and in the photon counting regime (details in [1]).
Each of the $n = 7$ measurement units induces a separation of 1.12 ± 0.02 pixels (px) along the x direction between the horizontal and vertical components of the polarisation of our single photons, corresponding to an interaction intensity of $\tilde{\epsilon} = 0.56 \pm 0.01$ px; this means that our observable eigenvalue spectrum spans over an interval of 7.84 ± 0.15 px.

II. DISTRIBUTIONS FOR ADDITIONAL RUNS

To complement the results for the additional runs (b)-(d), Fig. S 1 presents the corresponding distributions of clicks. Even if the photon spatial distribution at the end of the process is no longer a shifted Gaussian, but a multi-lobe structure, the expectation value of the pointer still provides the weak value of the pre- and postselected system.

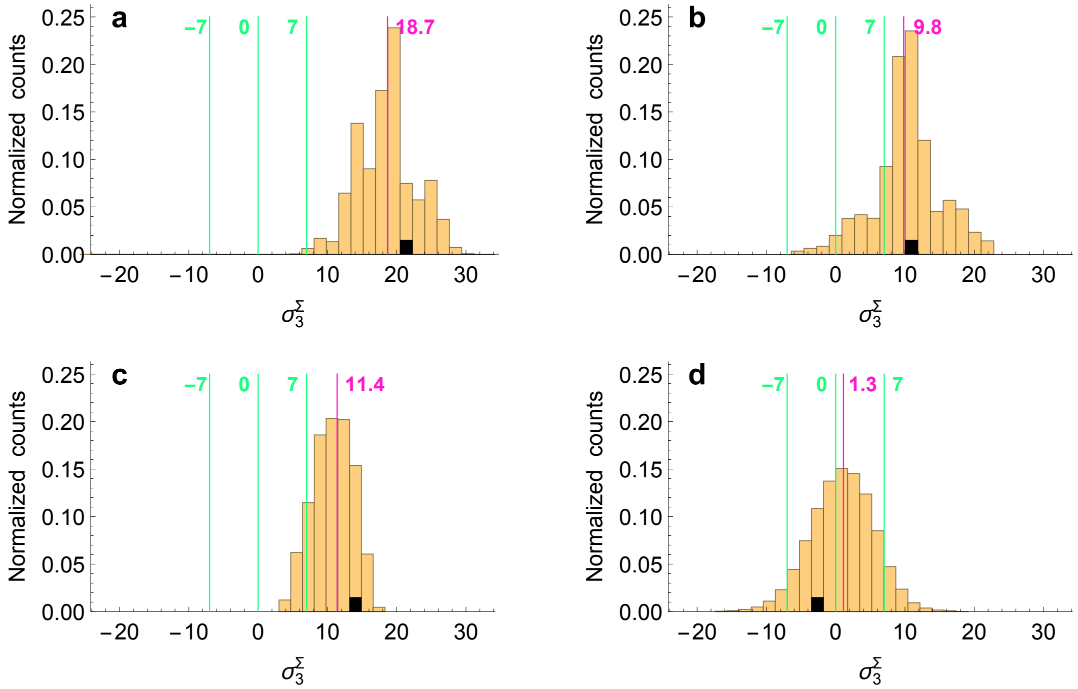

FIG. S 1: **Histograms of the photon counts.** Each plot shows the fraction of the total click events for each vertical array of pixels for the corresponding experimental run, see Table 1. The black square indicates the first click of the run, corresponding to the single-click experiment. The green lines indicate the borders and center of the spectrum. The purple line shows the expected (theoretical) weak value $(\sigma_3^\Sigma)_w$.

III. VARYING EXPERIMENTAL PARAMETERS

Table S I shows details of the four experimental runs reported in the manuscript. Together with the beam width $\tilde{\Delta}$ (note that $\Delta = \tilde{\Delta}/\tilde{\epsilon}$, see section I of the additional material), the reader can find the success probability of each experiment, i.e. the theoretical survival probability at the end of the whole measurement process (excluding losses due to imperfections of the optical elements involved, i.e. half-wave plates, birefringent crystals and polarisers), given by the formula

$$P^{(\mathrm{sur})} = \sum_{k,l=0}^{n} \binom{n}{k}\binom{n}{l} (\cos\alpha\cos\beta)^{(k+l)} (\sin\alpha\sin\beta)^{(2n-k-l)} e^{-\frac{(k-l)^2}{2\Delta^2}}, \tag{1}$$

as well as the number of counts N registered by our EM-CCD, dark counts subtracted. Regarding the dark counts subtraction, for each data set we tried two different approaches: the

#	1	2	3	4	5	6
	α	β	$\tilde{\epsilon}$ [px]	$\tilde{\Delta}$ [px]	$P^{(\mathrm{sur})}$	N
(a)	0.62	2.53	0.56 ± 0.01	3.27 ± 0.07	3.6×10^{-7}	31208
(b)	0.62	2.53	0.56 ± 0.01	1.78 ± 0.04	4.0×10^{-6}	131529
(c)	0.52	2.62	0.56 ± 0.01	1.65 ± 0.04	1.6×10^{-4}	221555
(d)	0.52	0.88	0.56 ± 0.01	1.73 ± 0.04	0.34	2.821×10^{7}

TABLE S I: **Initial parameters and acquisition data of the four experimental runs.** Columns 1-4 host the preparation parameters, and column 5 the survival probability $P^{(\mathrm{sur})}$ of each photon at the end of the robust weak measurement process. Column 6 shows the number of click events obtained in each experimental run (dark counts subtracted).

first one consisted on choosing a region of the EM-CCD camera not illuminated by the signal photons, evaluating the average pixel counts in that area and subtracting them from the pixels belonging to the photon detection region. The second one, instead, was based on fitting the photon counts distribution with a function composed of a (modified) two-dimensional Gaussian function plus a constant BIAS; the BIAS value obtained with this fit was then subtracted from the whole EM-CCD pixel array. The two approaches gave consistent results.

IV. UNCERTAINTY OF THE ESTIMATED WEAK VALUE IN THE SINGLE-CLICK EXPERIMENTS

The systematic uncertainties due to inhomogeneities in the birefringent crystals, that directly affect the uncertainty on Δ, were properly computed and included in the overall experimental uncertainty budget, and the same holds for the EM-CCD dark counts contribution.

Using Eq. (10) from the paper we can investigate the behavior of the reading uncertainty in the whole α and β parameters space (we found that the effect of the uncertainty on Δ is negligible in comparison). The full dependence of Δx on α and β is shown in Fig. S 2.

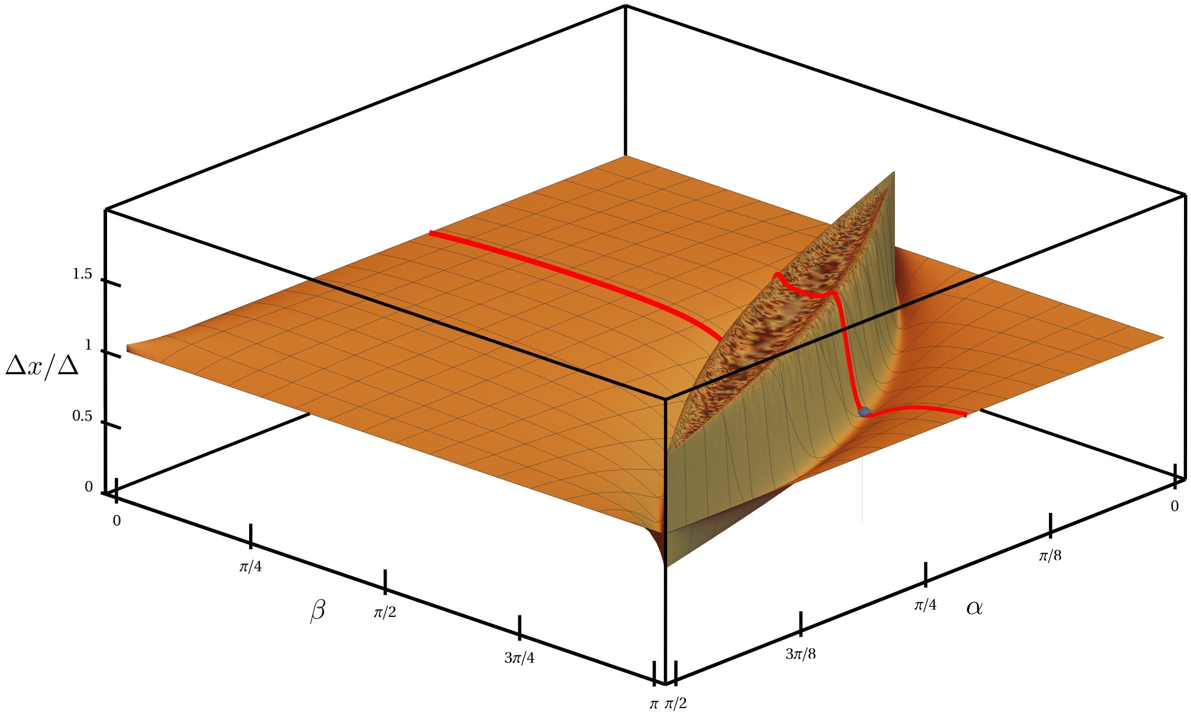

FIG. S 2: **Theoretically-predicted pointer uncertainty.** Predicted values for the final pointer uncertainty Δx for full parameter space of α and β (with $n = 7$ and $\Delta = 5.84$, like in experiment (a)), rescaled on Δ. The blue sphere denotes the intended settings $\alpha = 0.62$ and $\alpha = 2.53$, as well as the corresponding value for the uncertainty $\Delta x = 0.77\,\Delta$. The red line illustrates the cross-section taken for Fig. 3 in the paper, fixing $\alpha = 0.62$ and varying β.

It reaffirms the statement based on Fig. 3 in the main paper, i.e. that in the largest part of the parameters space the final uncertainty is close to the initial pointer width Δ. Only in the region where pre- and postselection become close to orthogonal one can observe an increase up to $1.6\,\Delta$ of the final uncertainty.

[1] A. Avella, I. Ruo-Berchera, I. P. Degiovanni, G. Brida, and M. Genovese, "Absolute calibration of an emccd camera by quantum correlation, linking photon counting to the analog regime", *Opt. Lett.* **41**, 1841-1844 (2016).
